# Description and performance of two diet quality scores based on the Nova classification

**DOI:** 10.11606/s1518-8787.2024058006470

**Published:** 2024-11-25

**Authors:** Caroline dos Santos Costa, Francine Silva dos Santos, Kamila Tiemann Gabe, Eurídice Martinez Steele, Fernanda Helena Marrocos-Leite, Neha Khandpur, Fernanda Rauber, Maria Laura da Costa Louzada, Renata Bertazzi Levy

**Affiliations:** I Universidade de São Paulo. Faculdade de Saúde Pública. Núcleo de Pesquisas Epidemiológicas em Nutrição e Saúde. São Paulo, SP, Brasil; II Universidade Federal de Ciências da Saúde de Porto Alegre. Departamento de Nutrição. Porto Alegre, RS, Brasil; III Universidade de São Paulo. Faculdade de Saúde Pública. Programa de Pós-graduação em Nutrição e Saúde. São Paulo, SP, Brasil; IV Universidade de São Paulo. Faculdade de Saúde Pública. Programa de Pós-graduação em Saúde Global e Sustentabilidade. São Paulo, SP, Brasil; V Wageningen University. Division of Human Nutrition and Health. Wageningen, The Netherlands; VI Universidade de São Paulo. Faculdade de Saúde Pública. Departamento de Nutrição. São Paulo, SP, Brasil; VII Universidade de São Paulo. Faculdade de Medicina. Departamento de Medicina Preventiva. São Paulo, SP, Brasil

**Keywords:** Eating, Diet Surveys, Surveys and Questionnaires, Validation Study

## Abstract

**OBJECTIVE:**

To describe two low-burden diet quality scores and evaluate their performance in reflecting the dietary share of the least and most processed foods defined within the Nova food system classification.

**METHODS:**

This cross-sectional study included data from the NutriNet-Brasil cohort. Participants answered the Nova24hScreener, a 3-minute self-administered questionnaire measuring the consumption of a set of foods on the day before. Food items included in this tool belong to two main groups of the Nova classification: unprocessed or minimally processed whole plant foods (WPF, 33 items) and ultra-processed foods (UPF, 23 items). Two scores were obtained by summing the number of items checked: the Nova-WPF and the Nova-UPF. We compared the scores, respectively, with the dietary intake (% of total energy) of all unprocessed or minimally processed whole plant foods and all ultra-processed foods obtained from a full self-administered web-based 24-hour recall performed on the same day.

**RESULTS:**

The approximate quintiles of each score had a direct and linear relationship with the corresponding % of energy intake (p-value for linear trend < 0.001). We found a substantial agreement between the intervals of each score and the corresponding % of energy intake (Nova-WPF score: Prevalence-Adjusted and Bias-Adjusted Kappa (PABAK) 0.72, 95%CI 0.64–0.81; Nova-UPF score: PABAK 0.79, 95%CI 0.69–0.88).

**CONCLUSIONS:**

These two scores performed well against the dietary share of unprocessed or minimally processed whole plant foods and ultra-processed foods in Brazil and can be used to evaluate and monitor diet quality.

## INTRODUCTION

Dietary patterns that prioritize the consumption of unprocessed or minimally processed whole plant foods – encompassing a diverse combination of fruits, vegetables, legumes, and whole grains, while limiting the intake of animal-source foods – along with the avoidance of ultra-processed foods (UPFs), have been recommended to improve both human and planetary health^1^. There is a large body of evidence on the benefits of higher consumption of unprocessed or minimally processed whole plant foods on the nutritional profile of diets, human health, and environmental sustainability^2,3,5^. At the same time, the dietary share of UPFs has been consistently associated with the overall deterioration of the nutrient profile of diets^6,7^ and a higher risk of several non-communicable diseases (NCDs)^8^.

The least and most processed food groups, as previously mentioned, belong to two of the four groups of the Nova classification, a framework that categorizes foods and beverages according to the extent and purpose of industrial processing they have undergone^1^. The first group comprises unprocessed and minimally processed foods that are subjected to processes that largely preserve the food matrix and do not involve the addition of salt, sugar, fat, or any other food substance^1^. Therefore, whole plant foods fall into this Nova group. The most processed group, on the other hand, comprises ultra-processed foods, which are industrial formulations consisting mostly of substances derived from foods and cosmetic additives (i.e. flavor enhancers, colors, emulsifiers, sweeteners, and thickeners)^1^.

Despite recommendations for healthy and sustainable diets, global evidence from repeated national food consumption and sales surveys suggests a shift away from traditional dietary patterns toward those based on animal-sourced foods and UPFs^12^. Evaluating and monitoring food consumption to track these global shifts in dietary patterns are essential. However, conventional tools, such as 24-hour recall or food frequency questionnaires, are not designed to collect dietary data aligned with food processing and require personnel and time resources that are not always available.

The current study presents the Nova24hScreener, a novel and practical 24-hour recall screener developed to easily assess the dietary intakes of two critical Nova groups in Brazil – the unprocessed or minimally processed whole plant foods and ultra-processed foods. Scores reflecting the intake level can be calculated using this screener. The screener consists of two lists of yes/no questions regarding the consumption of the most commonly consumed foods in Brazil, as determined by a nationally representative survey: one containing commonly consumed unprocessed or minimally processed whole plant foods grouped into 33 questions and the other containing ultra-processed foods grouped into 23 questions^20^.

Previous research has presented the ultra-processed foods list of the Nova24hScreener and tested the Nova-UPF score in a small convenience sample in a southeastern city in Brazil, indicating its potential to reflect the dietary share of ultra-processed foods^21^. Compared with other diet quality metrics, the Nova-UPF score has added a clearer value in terms of measuring the dimension of diets related to foods for which consumption should be limited/moderated^22^. On the other hand, the performance of the score for the unprocessed/minimally processed whole plant food component has not yet been evaluated, nor has the performance of the two scores in larger samples and across sociodemographic subgroups. The scores were proposed to represent two sub-constructs of the construct of diet quality based on food processing. The score based on unprocessed or minimally processed whole plant foods represents a dietary pattern based on foods with consistent literature on health benefits, whereas the Nova-UPF score represents a dietary pattern based on ultra-processed foods. Therefore, this study aimed to (1) describe two diet quality scores based on the Nova classification system, calculated using the Nova24hScreener, and (2) evaluate their ability to reflect the dietary share of unprocessed or minimally processed whole plant foods and ultra-processed foods using data from a large Brazilian study.

## METHODS

### Sample Selection

This is a cross-sectional analysis conducted with participants of the NutriNet-Brasil cohort, an ongoing web-based study that currently includes over 100,000 adult volunteers (18 years old or older) from all regions of Brazil, and aims to prospectively study the association between dietary patterns and chronic diseases. Recruitment started on January 26, 2020 and is largely based on multimedia campaigns. Participants self-registered on the project website (https://nutrinetbrasil.fsp.usp.br/) and, after consenting to participate, answered brief questionnaires about diet, health status, socioeconomic conditions, and other determinants of health every three months on the same website using a cellphone, tablet, or computer. The ethics committee of the School of Public Health from São Paulo University (process No. 88455417.8.0000.5421) approved the study.

For the current study, we recruited a quota-based subsample of NutriNet-Brasil participants (n = 1,800) to answer on the same day, first, the Nova24hScreener, from which two diet quality scores were obtained, and then a full 24-hour dietary recall. Recruitment was conducted at 6-month follow-up; all the NutriNet-Brasil participants who reached that point were considered eligible for the study. To achieve an even distribution of both sexes and individuals from all five Brazilian macro-regions, the invitations were randomly distributed across quotas (180 invitations for each sex in each of the five regions of the country). The number of invitations was three times the minimum expected sample size (n = 600), which was estimated based on a previous study that recommended a sample size of at least 50 to 100 subjects for each stratum when evaluating population subgroups in validation studies^23^ to account for exclusions or no responses due to possible cohort attritions. The recall data allow estimation of the dietary intake (% of total energy) of all unprocessed or minimally processed whole plant foods and all ultra-processed foods. Finally, among the participants who completed the Nova24hScreener (n = 894), we excluded those who did not complete the full recall (n = 65) or presented extreme energy intake (n = 17), i.e., total energy below the 1st percentile (676.7 Kcal) or above the 99th percentile (6,225.8 Kcal) of the total energy distribution in the recruited sample, resulting in a final sample of 812. Data collection was carried out over a two-month period (September and October 2020).

### Data Collection

The Nova24hScreener, a 3-minute self-administered questionnaire, asks participants about the intake of two lists of foods consumed on the day before (checkbox format, yes/no). The first list includes varieties of Nova unprocessed or minimally processed foods that are whole plant foods (grouped into 33 items) and the second, varieties of ultra-processed foods, including both plant-based and animal-based products (grouped into 23 items). Both lists are based on a similar tool used in national surveys in Brazil, whose development included pre-tests with users of a primary healthcare service^24^. The items included in the screener represent the most consumed unprocessed or minimally processed whole plant foods and ultra-processed foods in Brazil according to the national food consumption survey conducted by the 2008–2009 Household Budget Survey (POF) of the Brazilian Institute of Geography and Statistics^20^.

Unprocessed or minimally processed whole plant food items are grouped into six categories: fruits, excluding fruit juices (10 items); leafy vegetables (9 items); other vegetables, excluding roots and tubers such as potato and manioc (9 items); whole grains (3 items); legumes (1 item); and nuts (1 item). Ultra-processed foods items are grouped into three categories: beverages (6 items); ready-to-eat products created to replace meals (10 items); and products often consumed as snacks (7 items). The complete list of foods included in the Nova24hScreener is presented in Supplementary material^a^.

Although starchy vegetables such as potato and manioc are part of the Brazilian dietary pattern, particularly in specific geographical regions, the Nova-WPF score was based on food items with consistent literature on their protective effects on human health^1^. Therefore, these items were not included in the screener.

Two independent scores were obtained from the Nova24hScreener based on the simple sum of the checked items within each food group: the Nova score of Whole Plant Foods (Nova-WPF, ranging from zero to 33), and the Nova score of Ultra-Processed Foods (the Nova-UPF score, ranging from zero to 23) (Supplementary material^a^). Higher Nova-WPF scores represent healthier dietary patterns based on the Nova classification, as well as lower Nova-UPF scores. We used data collected through a full and validated self-administered web-based 24-hour recall to evaluate the performance of the Nova-WPF and the Nova-UPF scores against the % of total energy from all unprocessed or minimally processed whole plant foods (excluding roots and tubers) and all ultra-processed foods, respectively. The web-based 24-hour recall was specifically designed to capture the consumption of each of the four Nova food groups (hereinafter called Nova24h)^25^. In the Nova24h, participants reported all foods and drinks consumed on the day before and their respective amounts based on a predetermined list of foods and standardized categories. Nova24h was previously validated against a standard interviewer-led multiple-pass 24-hour dietary recall. Description and validation details are explained in a previously published paper^25^.

Briefly, the food list integrated into the Nova24h was developed using nationally representative food consumption data and the food grouping structure of the Brazilian Household Budget Survey 2008-2009^20^. The review comprises 57 key questions, and detailed information is also requested regarding food source, preparation method, and additions (other foods or culinary ingredients added to preparations). All possible variations from the key questions are listed in the supplementary material of the Nova24h methodological paper. There are a total of 526 food items or combinations of responses to the key questions, including 347 individual or grouped items (e.g. ‘whole milk’ or ‘squash, zucchini or eggplant’) and 179 culinary preparations, which are subsequently disaggregated into underlying ingredients (e.g. ‘cooked rice’ is disaggregated into: rice, oil, onion, garlic, and salt). All food items and ingredients from the recipes were linked with the Brazilian Table of Food Composition 7.0 (TBCA) as the primary source or with the United States Department of Agriculture (USDA) National Nutrient Database^26,27^ to obtain energy, macronutrient, and micronutrient content. Each item is also classified using a standard method according to the extent and purpose of industrial food processing established by the Nova system into four groups: unprocessed or minimally processed foods, processed culinary ingredients, processed foods, and ultra-processed foods^28^.

The full 24-hour recall provided data for calculating the percentage (%) of energy contribution from unprocessed or minimally processed whole plant foods (including all fruits, vegetables, whole grains, legumes, and nuts) and the percentage (%) of energy contribution from all ultra-processed foods. In sequence, we compared the Nova-WPF and Nova-UPF scores with their respective percentage (%) of energy contribution.

Sociodemographic information was collected in the recruitment of the participants, including geographic region of the country (North, Northeast, Southeast, South, Mid-West), sex (male, female), schooling level (0–11, 12+, in years), and age range (18–34, 35–59, 60+, in years).

### Statistical Analysis

First, we described the prevalence (%) of consumption on the day before the interview for each food item included in the Nova24hScreener and the distribution of the Nova-WPF and Nova-UPF scores. Then, we presented the mean dietary share (% of total energy intake with 95% CI) of the unprocessed and minimally processed whole plant foods and ultra-processed foods obtained from the full 24-hour recall according to the non-overlapping intervals of each score (0–4, 5–6, 7, 8–9, 10+ for the Nova-WPF score; and 0, 1, 2, 3, 4+ for the Nova-UPF score), using linear regression models to analyze the linear trend.

The above-mentioned intervals of each score correspond to the approximate quintiles generated from the scores in their counting format. In fact, we first intended to generate quintiles for the Nova scores. However, considering the nature of repetitions in counting variables, when generating quintiles using the *xtile* command from Stata, the distribution of participants in each of the five categories did not exactly correspond to 20%, as expected in a uniform data distribution. Considering the unequal distribution of participants across the intervals of the scores, we chose to replicate the marginal proportions of these intervals to categorize participants according to their 24-hour recall dietary share of whole plant or ultra-processed foods to obtain comparable groups. In this way, the distribution of the dietary share of unprocessed or minimally processed whole plant foods was divided into five parts by applying the same proportions of the Nova-WPF score to approximate quintiles. The same was done for the dietary share of ultra-processed foods based on the proportions of the Nova-UPF score that approximated quintiles. For example, if 30%, 15%, 20%, 15%, and 20% of the participants were part of the first, second, third, fourth, and fifth intervals of a score, respectively, we would replicate these proportions for the first to the fifth intervals of the reference measure. Thus, from now on, we refer to these categories as “approximate” quintiles or intervals.

To evaluate the agreement between approximate quintiles of the scores and respective reference measures, we compared individuals’ classification according to Nova-WPF score intervals with the classification according to intervals of the dietary share (% of the total energy) of whole plant foods. Likewise, we compared the classification of individuals according to the Nova-UPF score intervals with that according to the intervals of the dietary share (% of the total energy) of UPF. We calculated the PABAK (Prevalence-Adjusted and Bias-Adjusted Kappa) to estimate the agreement between the classifications based on the Nova-WPF or Nova-UPF scores and the ones based on the percentage of total energy from whole plant or ultra-processed foods, respectively. This index is a modification of the kappa statistic that adjusts for prevalence and bias. While kappa is highly sensitive to the prevalence of the condition, PABAK depends only on the observed agreement and is particularly useful when data are imbalanced, where one category is more prevalent than others^29^. All agreement analyses were replicated for each socioeconomic strata (geographic region, sex, schooling level, and age range) to confirm the performance of these subgroups. Values greater than 0.80 indicate an almost perfect agreement; between 0.61 and 0.80, a substantial agreement; between 0.41 and 0.60, moderate; between 0.21 and 0.40, fair; and equal to or less than 0.20, slight^30^.

All analyses were performed using the Stata^®^ statistical package, version 16.1 (StataCorp. 2019. Stata Statistical Software: Release 16. College Station, TX: StataCorp LLC), except for agreement analyses, which were performed in RStudio using the statistical package “irrCAC” where the 95% confidence intervals were also calculated using the matrix’s square weights^31^.

## RESULTS

A total of 812 NutriNet-Brasil participants answered the Nova24hScreener and were included in the analysis. As planned, they were similarly distributed across sexes and the five Brazilian regions. Participants included young (18–34 y, 36.6%), middle-aged (35–59 y, 51.6%), and older (60y+, 11.8%) adults. Similar to what is observed in the total sample of the Nutrinet-Brasil cohort, most participants (85.6%) had university education (Supplementary material^a^).

**Figure 1:**
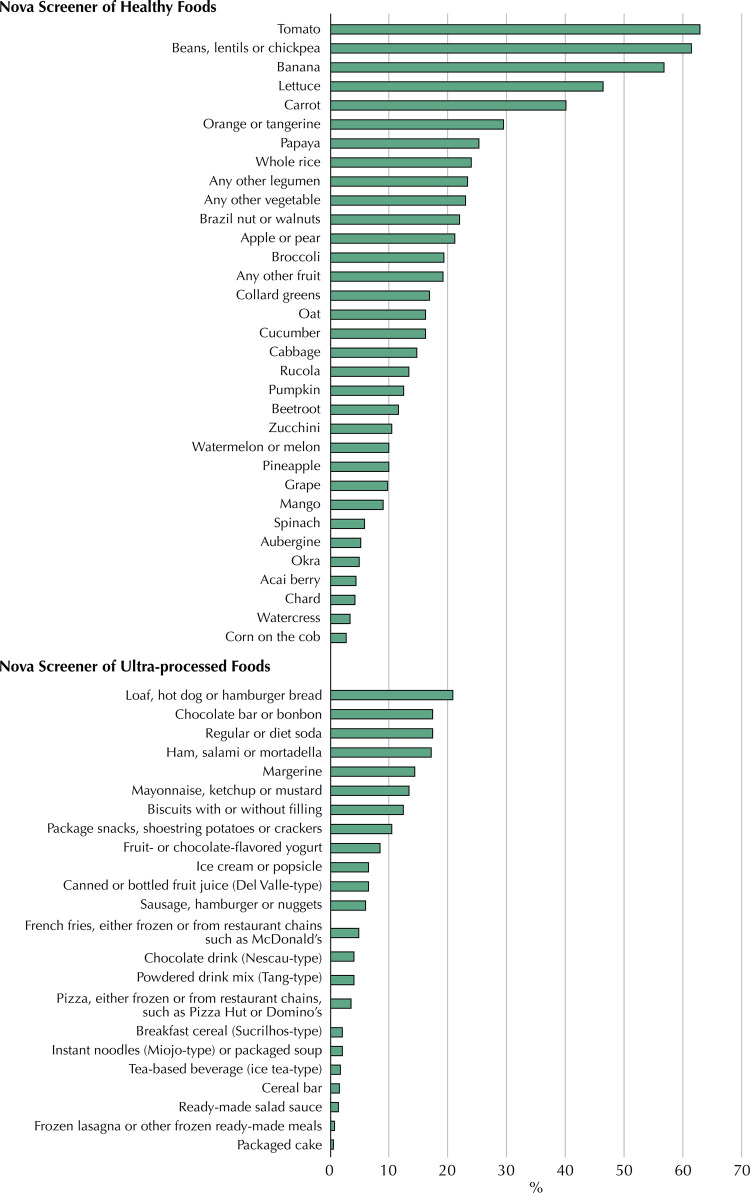
Proportion (%) of consumption of the food items included in the Nova24hScreener on the day before. Participants (n = 812) from the NutriNet-Brasil cohort (2020).

Among the unprocessed or minimally processed whole plant foods included in the Nova-WPF score, tomatoes, beans/lentils/chickpeas, bananas, lettuce, and carrot were the five most frequently consumed items. Over 40% of participants consumed each of these foods on the previous day. Conversely, bread, chocolate bars, soda, reconstituted meat products, and margarine, each consumed by 15%–20% of participants the day before, were the most frequently consumed ultra-processed items (Figure 1).

**Figure 2:**
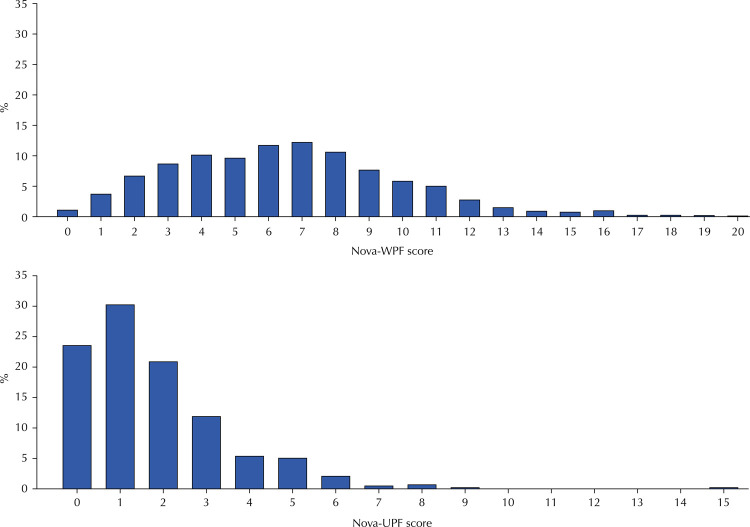
Distribution of the Nova-WPF score and the Nova-UPF score. Participants (n = 812) from the NutriNet-Brasil cohort (2020).

Note: The Nova-WPF score (Nova score of Whole Plant Foods) ranged from 0 to 33, and the Nova-UPF score (Nova score of Ultra-Processed Foods) ranged from 0 to 23.

The Nova-WPF score had a nearly normal distribution, ranging from 0 to 20 food items consumed the day before. Almost one-fifth of the participants (18.1%) scored 10+ (highest interval). The Nova-UPF score distribution ranged from 0 to 15 and was right-skewed. Almost one-fourth of the participants (23.5%) scored null, 30.2% scored one, 20.8% scored two, while 13.7% scored 4+ (highest interval) (Figure 2).

**Figure 3:**
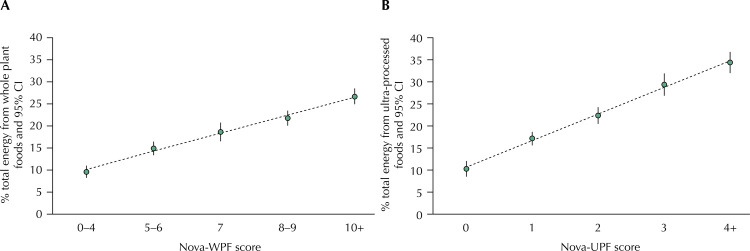
Mean dietary share (% of total energy intake with 95%CI) of (a) whole-plant foods obtained from the full 24-hour recall according to Nova-WPF score intervals and (b) ultra-processed foods obtained from the full 24-hour recall according to Nova-UPF score intervals. Participants (n = 812) from the NutriNet-Brasil cohort (2020).

Figure 3 shows that the dietary share of all unprocessed or minimally processed whole plant foods (% of total energy intake from these foods, estimated by the full 24-hour recall) increased linearly with the increase in the intervals of the Nova-WPF score (p-value for linear trend < 0.001). At the same time, the dietary share of ultra-processed foods (% of total energy intake from these foods, estimated by the full 24-hour recall) increased linearly with the increase in the intervals of the Nova-UPF score (p-value for linear trend < 0.001).

Nova-WPF score: Nova score of Whole Plant Foods; Nova-UPF score: Nova score of Ultra-Processed Foods.

p-value for linear trend < 0.001 in all associations.

There was a substantial agreement between the distribution of participants according to the intervals of the Nova-WPF score and the corresponding intervals of the dietary share of unprocessed or minimally processed whole plant foods (PABAK of 0.72; 95%CI 0.64–0.81). A substantial agreement was also observed between intervals of the Nova-UPF score and corresponding intervals of the dietary share of ultra-processed foods (PABAK of 0.79; 95%CI 0.69–0.88) (Tables 1 and 2). Similar agreements were observed in all sociodemographic strata (geographic regions of the country, sex, schooling level, and age range; Table S1 in Supplementary material^a^).

**Table 1:** Agreement between participants classification according to Nova-WPF score intervals, estimated using the Nova24hScreener, and corresponding intervals of the dietary share of whole plant foods, estimated using a full 24-hour dietary recall. Participants (n = 812) from the NutriNet-Brasil cohort (2020).

% of total energy intake from whole plant foods (full 24h recall)	Nova-WPF score intervals (Nova24hScreener)						PABAK (95%CI)
	0–4	5–6	7	8–9	10+	Total	
≤ 9.67	17.2	7.6	1.8	2.0	1.5	30.1	0.72 (0.64–0.81)
9.68–15.35	6.8	4.8	3.6	4.1	2.1	21.4	
15.36–19.34	2.3	3.1	1.8	2.7	2.2	12.1	
19.35–27.40	2.8	3.1	3.2	4.8	4.3	18.2	
≥ 27.41	1.0	2.7	1.7	4.7	8.0	18.1	
Total	30.1	21.3	12.1	18.3	18.1	100.0	

PABAK: Prevalence-Adjusted and Bias-Adjusted Kappa; Nova-WPF score: Nova score of Whole Plant Foods.

**Table 2:** Agreement between participant classification according to Nova-UPF score intervals, estimated using the Nova24hScreener, and corresponding intervals of the dietary share of ultra-processed foods, estimated using a full 24-hour dietary recall. Participants (n = 812) from the NutriNet-Brasil cohort (2020).

% of total energy intake from ultra-processed foods (full 24h recall)	Nova-UPF score intervals (Nova24hScreener)						PABAK (95%CI)
	0	1	2	3	4+	Total	
≤ 8.77	12.6	8.3	2.0	0.4	0.4	23.7	0.79 (0.69–0.88)
8.78–19.55	7.1	10.5	8.1	2.5	2.1	30.3	
19.56–28.37	2.2	5.7	5.2	4.4	3.2	20.7	
28.38–37.85	1.0	3.4	3.6	1.8	2.0	11.8	
≥ 37.86	0.6	2.3	2.0	2.7	6.0	13.6	
Total	23.5	30.2	20.9	11.8	13.7	100.0	

PABAK: Prevalence-Adjusted and Bias-Adjusted Kappa; Nova-UPF score: Nova score of Ultra-Processed Foods.

## DISCUSSION

This study aimed to describe two diet quality scores based on the Nova classification system and evaluate their performance in reflecting the dietary share of unprocessed or minimally processed whole plant foods and ultra-processed foods. The Nova-WPF and Nova-UPF scores, obtained from a simple 3-minute screener, presented significant direct and linear relationships with the dietary share of unprocessed or minimally processed whole plant foods and ultra-processed foods, respectively, assessed using a validated self-administered web-based 24-hour recall. Substantial agreement was found between participants classification according to the intervals of each score and the corresponding intervals of the dietary share of the foods included in each score.

A previous study using a convenience sample of 300 individuals from São Paulo, Brazil, evaluated the performance of the Nova-UPF score in reflecting the % of total energy from ultra-processed foods and found a substantial agreement with the reference measure^21^. Our study adds to the literature by confirming this performance in a larger and more diverse sample across geographic regions, sex, schooling level, and age groups. This study is also the first to evaluate the performance of the unprocessed or minimally processed whole plant foods score in reflecting the dietary share of unprocessed or minimally processed whole plant foods. Both scores were tested for their ability to predict BMI gain in another study. The Nova-UPF and Nova-WPF scores were independently and linearly associated with mid-term higher and lower risks of BMI gain, respectively^32^.

Regarding the Nova-WPF score, it is noteworthy that the first group of the Nova classification refers to a range of unprocessed or minimally processed foods – including animal-sourced unprocessed or minimally processed foods. Although the intake of this whole Nova group was associated with a better dietary nutritional profile^5^, we included in the score only the food groups that consistently prevented NCDs^2,3,33^. Furthermore, the scores as proposed in the present study are in line with the current dietary recommendations, such as the Planetary Health Diet and the Dietary Guidelines for the Brazilian population, because they allow monitoring and evaluating the consumption of foods that are encouraged and those that are recommended to avoid or limit^2,34^.

Although we found substantial agreement in ranking individuals according to the intervals of the % of total energy from both food groups, it is important to highlight that the agreement between the scores and the reference measure was higher in the extreme intervals. The low variability of the scores may have led to lower accuracy in classifying individuals in the intermediate categories because a change in the classification can occur even with an increase or decrease in only one point of the score, and the probability of classification bias in intermediate categories is higher than in extreme categories. Particularly for the Nova-UPF score, most of the population scored only up to 3 of the 23 points, which is consistent with the findings of previous studies using the same or a similar score^21,35,36^. However, both the low consumers, according to the Nova-WPF score (those with the lowest consumption of unprocessed or minimally processed whole plant foods), and the high consumers, according to the Nova-UPF score (those with the highest consumption of ultra-processed foods), showed better agreement than the intermediate categories, precisely those that represent the most critical population subgroups when aiming to provide information to policymakers. Moreover, we found a dose-response relationship between the scores’ intervals and the reference measure, suggesting fair discriminatory power when used as a continuous measurement.

According to the Healthy Diets Monitoring Initiative, several metrics have been developed to measure healthy diets, but only four—Global Diet Quality Score (GDQS); Minimum Dietary Diversity for Women (MDD-W); Nova ultra-processed foods (UPF) score (Nova-UPF score); and Global Dietary Recommendations (GDR) score—were considered by the expert group as having relative advantages to meet needs and reflect the specific sub-constructs of relevance, including nutrient adequacy, macronutrient balance, diversity, and moderation^22^. Among these metrics, the Nova-UPF score added a clearer value, i.e. a more specific, precise, and interpretable tool, in measuring the component of diets related to limited intake of unhealthy foods, particularly ultra-processed foods, which are explicitly associated with risks of NCDs (sub-construct of moderation)^22^. The Nova-WPF score would benefit from further comparisons with existing metrics such as the GDQS and GDR scores.

We are aware that our study has limitations. Our sample was mostly composed of participants with high schooling levels, most likely because the study was fully conducted online and the questionnaires were self-reported. However, a review of food consumption validation studies recommended a sample size of at least 50 to 100 subjects for each stratum when evaluating population subgroups^23^, which was achieved even for low-schooled participants. In addition, the same source of error could affect the scores and reference measures because they are both self-reported. Recall bias or intentional underreporting of some food items is also possible for both scores and reference measures. Considering this scenario, it would be relevant to validate the performance of the scores in predicting health outcomes in future studies, as previously done for BMI gain^32^. The reference measure (Nova24h, the full recall) was filled in immediately after the Nova24hScreener. In validation studies, it is mandatory that the tool under validation is completed first, before the method of reference, and considering the aim of our study, both tools had to be completed on the same day (as they related to a 24h recall period). This method could raise concerns about possible inflation or overestimated agreement considering that the foods asked in the Nova24hScreener could be more easily remembered when completing both instruments on the same day. However, the full recall (Nova24h) asked about the consumption of a much larger number of food items (n = 526) than the screener (n = 56)^25^. As strengths of the study, we highlight the careful methods, including quota-based recruitment according to sex and macro-region, and the application of a previously validated instrument to obtain the reference measures (the Nova24h full recall). Additionally, our study confirmed the performance of the Nova-UPF score in a larger sample, as well as demonstrated the performance of the unprocessed/minimally processed whole plant food component, namely the Nova-WPF score.

These two scores that (a) capture the most consumed unprocessed or minimally processed whole plant foods and ultra-processed foods, (b) present a good performance against 24-hour recall measures of food consumption based on the Nova classification system, and (c) are calculated using data from a low research burden screener (low cost, rapid application, and straightforward to analyze) can facilitate measuring and monitoring of the adherence to dietary patterns associated with NCDs. The increasing dominance of ultra-processed foods in the current food environment at the expense of unprocessed or minimally processed whole foods and the likely related increase in the risk of NCDs and all-cause mortality contribute to the relevance of the Nova24hScreener and its scores, allowing for the collection of information that can guide policies and actions in public health. Finally, even though the Nova24hScreener and its scores were developed based on the Brazilian context, they can be easily adapted for use in or validated to other countries (as it is currently happening in Ecuador, India, and Senegal)^37,38^. To check for the appropriateness of the screener items and select context-specific examples within each item, countries could use data from national dietary intake surveys or purchase surveys and/or other methodologies. In this context, the screener represents a possible tool to be introduced into monitoring and evaluation systems around the world.

## Footnotes

a Supplementary material is available at: https://osf.io/dxau6/
